# Patient characteristics and prognostic factors in advanced treatment lines in metastatic neuroendocrine tumors

**DOI:** 10.1007/s40618-026-02857-9

**Published:** 2026-03-31

**Authors:** Philipp Melhorn, Genti Zhitia, Markus Raderer, Barbara Kiesewetter

**Affiliations:** Department of Medicine I, Division of Oncology, Medical University of Vienna, Waehringer Guertel 18 – 20, Vienna, A-1090 Austria

**Keywords:** Neuroendocrine neoplasms, Prognosis, Survival analysis, Outcome

## Abstract

**Background:**

Given the biological heterogeneity of neuroendocrine tumors (NET), several prognostic factors such as morphology, primary site, and certain blood markers have been identified. However, their prognostic significance in the context of advancing treatment lines and late-stage disease is unclear.

**Methods:**

In this retrospective monocentric study, patient characteristics including blood-based inflammation indices at initiation of different lines of therapy (LoT) were assessed, and potential prognostic factors were evaluated with regard to overall survival (OS) from LoT start.

**Results:**

A total of 254 patients with NET G1/G2 (74%), NET G3 (8%), or typical/atypical carcinoid (18%) were included. All tumors were metastatic at treatment start, originating from the small intestine (36%), pancreas (28%), lung (17%), or other sites (19%). All patients had at least one palliative systemic therapy, the most frequent initial LoT was somatostatin analogs (SSA, *n* = 160, 63%), the most common second/third line peptide receptor radionuclide therapy (PRRT, *n* = 79 or 48% and *n* = 20 or 27%, respectively). Median OS from first-line start was 84.3 months (*n* = 250), and 48.6 months (*n* = 162) and 37.2 months (*n* = 75) from second- and third-line start, respectively. Patients reaching later LoT were more likely to have pancreatic NET with higher (≥ 10%) Ki-67 values. In univariate analysis, elevation of alkaline phosphatase (ALP) and chromogranin A (CGA) were negative prognostic factors in the first and second LoT subpopulation. Blood-based inflammation indices showed mixed prognostic value.

**Conclusion:**

Patients at the start of an early versus later LoT had different clinical characteristics, treatment patterns, and prognosis. These insights might help to refine prognosis estimates.

**Graphical abstract:**

**Figure:**
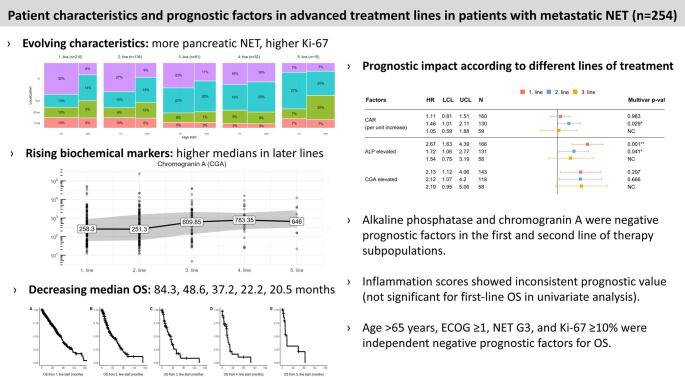

## Introduction

Neuroendocrine tumors are a relatively rare malignant disease with a comparably favorable prognosis given their generally slow growth rate [1]. As defined in the most recent World Health Organization (WHO) classification, this clearly sets them apart from neuroendocrine carcinomas (NEC), which are poorly differentiated and clinically very aggressive [2]. The outcome can vary considerably as a result of their significant heterogeneity in terms of tumor biology, localization of the primary tumor, and endocrine activity [3]. Hence, it seems important to further characterize individual patient risk factors that may guide clinical management. As comprehensively discussed in a recent guidance document, there are several prognostic and treatment-informing biomarkers in NET that may be useful in the context of patient management, albeit their evidence level is mostly poor [4]. The somatostatin receptor (SSTR) represents a classical theragnostic target used in NET for both positron emission tomography (PET) imaging and radioligand therapy (PRRT), but the lack of recurrent molecular alterations makes it challenging to tailor treatments to individual patients as part of a precision medicine approach [5].

According to data from the Surveillance, Epidemiology, and End Results (SEER) program of the United States [6], the Netherlands Cancer Registry [7], and the National Cancer Registry of Spain [8], male sex, older age, high tumor grade, advanced stage, and certain tumor sites represent indicators of poor prognosis in digestive NET. Many other studies involving NET of various origins have shown a prognostic value of additional clinical factors, including Eastern Cooperative Oncology Group (ECOG) performance status [9], Ki-67 proliferation index [10], surgical resection [11], uptake on SSTR imaging [12], and endocrine activity [13]. Furthermore, elevation of tumor markers like CGA [14] or neuron-specific enolase (NSE) [15] and of other parameters like ALP [16] is linked to an unfavorable prognosis. Finally, blood-based indices of systemic inflammation (e.g., neutrophil–to–lymphocyte ratio) [17] or of both inflammation and nutritional status (e.g., C-reactive protein–albumin ratio) [18] have also been associated with worse outcome. Based on such clinical features, prognostic scores have been proposed for NET [19–22], but the relevance in clinical practice has been limited.

In most of these studies, the prognostic value was determined at a specific point early in the course of the disease, e.g., at diagnosis or treatment start. Apart from that, analyses of prognostic factors in clinical trials might provide insights for pretreated patients or with regard to specific treatments [23–25]. However, there currently seems to be no systematic evaluation of clinical characteristics and prognostic factors in patients who are progressing through the various LoT that NET patients may receive over the course of the disease.

Since real-world data on advanced disease stages in NET are generally lacking, it is unclear to what extent the composition of patient subpopulations changes and how this could affect survival estimates. Consequently, it might be of practical interest to continuously update and adjust the prognosis estimate of NET patients, e.g., by using the most recent blood values at new LoT start for prognostication. Thus, the primary purpose of this retrospective study was to investigate potential differences in patient characteristics and prognosis depending on the different treatment lines in NET. Furthermore, we aimed to systematically describe the landscape of treatment lines in NET, and we wanted to investigate the prognostic role of blood-based inflammation indices and tumor markers in this context.

## Methods

This study is a retrospective data analysis of patients with NET of grades G1-G3 and diverse primary origin (such as gastroenteropancreatic, pulmonary, and other sites) who were metastatic and received treatment at the Medical University of Vienna, a European Neuroendocrine Tumor Society (ENETS) certified Center of Excellence, between January 2010 and September 2023. Exclusion criteria were NEC histology, no distant metastases, no systemic palliative treatment, and lack of clinical or laboratory data. Routinely documented patient data were abstracted from electronic medical records and entered into a secure database created with FileMaker (Claris International Inc., Santa Clara, CA, USA). Predefined extracted parameters included baseline patient data (e.g., age, sex, and ECOG performance status), biochemical values (e.g., blood cell counts and tumor markers), and cancer characteristics (e.g., tumor stage, primary localization, grade, and Ki-67 index). Furthermore, treatment and outcome data (e.g., tumor resection, LoT, treatment duration, and progression / survival status) were extracted. This study had received approval from the Ethics Committee of the Medical University of Vienna (EK number 1852/2023); obtaining specific informed consent from patients for study inclusion was not necessary.

### Study objectives

The main objective was to calculate hazard ratios (HR) and 95% confidence intervals (CI) for clinical factors and routinely assessed blood markers in Cox regression analysis of OS from first-line therapy start as well as from the start of subsequent LoT. A further objective was to descriptively analyze the patient subsets that had at least one, two, three, four, or five treatment lines (here termed LoT subpopulations).

### Definitions

OS from treatment start was defined as the period from the initiation date of the respective LoT to date of death or to last patient follow-up date in case of censoring. LoT was defined as the number of palliative-intent systemic anti-tumor treatments, excluding add-on therapies, maintenance therapies, and dose changes. Patients were switched to next-line treatment in case of tumor progression, as established by routine radiologic re-assessment and routine discussions in our multi-disciplinary tumor board. Inflammation indices included the C-reactive protein–albumin ratio (CAR), the neutrophil–to–lymphocyte ratio (NLR), the platelet–to–lymphocyte ratio (PLR), the monocyte–to–lymphocyte ratio (MLR), and the systemic inflammation response index (SIRI, = monocytes x neutrophils / lymphocytes), as previously described [18, 26]. The unit used for C-reactive protein was mg/l, for albumin g/l, and for cell counts G/l. Only laboratory values obtained less than 90 days before the start of treatment were considered. Depending on the laboratory, the reference values differed slightly, with an elevation in ALP generally defined as > 130 U/l (male) or > 105 U/l (female) and with elevated CGA most commonly specified as > 76.3 ng/ml and increased NSE as > 16.3 µg/l.

### Statistical analysis

The statistical programming language R version 4.5.1 (R Foundation for Statistical Computing, Vienna, Austria) with the packages tidyverse [27], gtsummary [28], gt [29], and cowplot [30] was employed for statistical analysis. Quantitative variables were presented as medians and ranges, whereas categorical variables were given as percentages and frequencies. Treatment frequencies in the respective lines of therapy were visualized with the package waffle [31]. Patient characteristics for the different subsets at therapy start were shown in a mosaic plot created with ggmosaic [32]. Forest plots were customized to compare the HR of the different LoT subpopulations using the package forestplot [33]. OS curves were generated with the Kaplan-Meier method using ggsurvfit [34]. Univariate and multivariable Cox proportional hazards regression models were fitted using the package survival [35]. Blood markers with clear reference ranges (i.e., ALP, CGA, and NSE) were treated as binary variables, whereas inflammation indices were modeled as continuous variables. The proportional hazards assumption was assessed statistically and graphically, and the linearity assumption was checked visually for continuous variables. For the LoT subpopulations, a multivariable-analysis p-value was calculated by adjusting for age, ECOG, and grading, unless < 10 events per variable (EPV) were observed. This was an exploratory and hypothesis-generating study with no adjusting for multiple testing. The sample size resulted from the total number of patients available for analysis. Consequently, a formal sample size or power calculation was not performed.

## Results

### Characteristics of patients at diagnosis

In total, 254 patients with histologically confirmed NET were included in this analysis, see Table 1. The study population comprised 119 female (47%) and 135 male (53%) patients. At diagnosis, 35% were over the age of 65 years, with the median age being 61 years and the range 21–83 years. Most patients had unimpaired performance status at diagnosis (ECOG 0 in 89%). Almost two-thirds (64%) had primary tumor resection.

The most frequent primary site was the small intestine (*n* = 92, 36%), followed by pancreas (*n* = 71, 28%), lung (*n* = 43, 17%), and other origin (*n* = 48, 19%). In terms of grading, the majority (74%) was NET G1/G2 (*n* = 187, comprising 60 NET G1, 107 NET G2, and 20 NET G1/G2 unclassified), 8% had NET G3 (*n* = 21), 7% (*n* = 18) were typical and 11% (*n* = 28) atypical carcinoids. The median Ki-67 index was 5% (range: 0–60), one-third (34%) overall and half (52%) of pancreatic NET had Ki-67 ≥ 10%. Functional symptoms were documented in 31% of patients. At diagnosis, 75% had distant metastatic disease, with differences based on localization (88% in small intestinal NET versus 49% in lung NET). All patients were metastatic at treatment start.

**Table 1:** General patient characteristics by primary tumor localization

Characteristic	*N*	Overall *N* = 254^*1*^	Pancreas *N* = 71^*1*^	Small intestine *N* = 92^*1*^	Lung *N* = 43^*1*^	Other *N* = 48^*1*^
**Sex**	254					
Female		119 (47%)	31 (44%)	41 (45%)	24 (56%)	23 (48%)
Male		135 (53%)	40 (56%)	51 (55%)	19 (44%)	25 (52%)
**Age at diagnosis**	254	61 (21, 83)	54 (21, 81)	63 (29, 82)	68 (31, 83)	59 (38, 78)
Over 65 years	254	89 (35%)	14 (20%)	36 (39%)	24 (56%)	15 (31%)
**ECOG performance score**	216					
0		193 (89%)	55 (93%)	79 (92%)	30 (81%)	29 (85%)
1		20 (9.3%)	3 (5.1%)	6 (7.0%)	7 (19%)	4 (12%)
2		2 (0.9%)	1 (1.7%)	1 (1.2%)	0 (0%)	0 (0%)
3		1 (0.5%)	0 (0%)	0 (0%)	0 (0%)	1 (2.9%)
**Surgery**	254	162 (64%)	32 (45%)	83 (90%)	25 (58%)	22 (46%)
**Histology**	254					
NET G1		60 (24%)	11 (15%)	39 (42%)	0 (0%)	10 (21%)
NET G2		107 (42%)	38 (54%)	42 (46%)	0 (0%)	27 (56%)
NET G3		21 (8.3%)	11 (15%)	5 (5.4%)	0 (0%)	5 (10%)
TC		18 (7.1%)	0 (0%)	0 (0%)	17 (40%)	1 (2.1%)
AC		28 (11%)	0 (0%)	0 (0%)	26 (60%)	2 (4.2%)
NET G1/G2		20 (7.9%)	11 (15%)	6 (6.5%)	0 (0%)	3 (6.3%)
**Ki-67 index (%)**	210	5 (0, 60)	10 (1, 60)	3 (1, 31)	5 (0, 30)	9 (1, 40)
Ki-67 ≥ 10%	210	72 (34%)	29 (52%)	13 (16%)	11 (33%)	19 (48%)
**Functional symptoms**	241	75 (31%)	10 (16%)	51 (56%)	6 (14%)	8 (17%)
**Disease setting at diagnosis**	247					
Localized		37 (15%)	6 (9.0%)	3 (3.4%)	18 (42%)	10 (21%)
Locally advanced		24 (9.7%)	9 (13%)	8 (9.0%)	4 (9.3%)	3 (6.3%)
Distant metastases		186 (75%)	52 (78%)	78 (88%)	21 (49%)	35 (73%)
**Distant metastasis at diagnosis**	248	186 (75%)	52 (76%)	78 (88%)	21 (49%)	35 (73%)
Distant lymph nodes	239	55 (23%)	15 (24%)	19 (22%)	8 (20%)	13 (28%)
Liver	244	161 (66%)	46 (71%)	69 (77%)	10 (24%)	36 (75%)
Bone	243	38 (16%)	8 (12%)	11 (12%)	11 (27%)	8 (17%)
Lung	241	14 (5.8%)	1 (1.5%)	1 (1.1%)	8 (20%)	4 (8.5%)
Brain	239	2 (0.8%)	0 (0%)	0 (0%)	2 (4.9%)	0 (0%)
Other	254	13 (5.1%)	1 (1.4%)	6 (6.5%)	3 (7.0%)	3 (6.3%)
Local lymph nodes	232	144 (62%)	30 (52%)	81 (92%)	15 (37%)	18 (40%)
**Metastatic at treatment start**	254	254 (100%)	71 (100%)	92 (100%)	43 (100%)	48 (100%)

^*1*^n (%); Median (Min, Max). Other localizations included 23 cancers of unknown primary (CUP), 6 duodenal, 5 rectal, 4 gastric, 4 colon, 3 thymic, and 3 presacral NET. Functional symptoms were due to hormone overproduction of serotonin (*n* = 62), insulin (*n* = 4), gastrin (*n* = 3), parathyroid hormone-related peptide (*n* = 2), adrenocorticotropic hormone (*n* = 2), calcitonin (*n* = 1), and glucagon (*n* = 1).

### Description of treatment lines

**Fig. 1:**
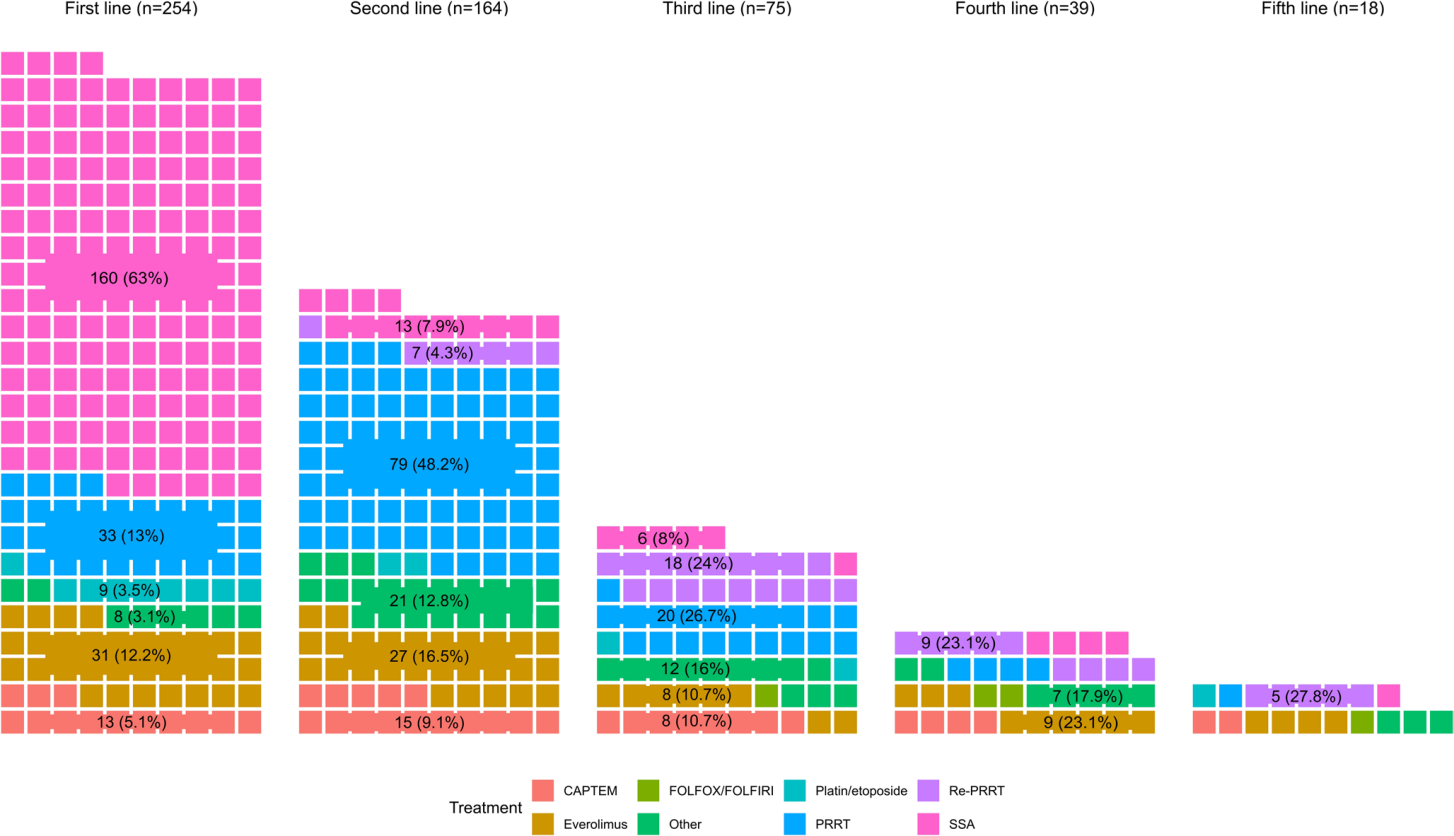
Number of therapies that were applied in the different treatment lines (each block represents a patient)

All 254 NET patients received at least one line of palliative systemic treatment, of whom the treatment start date was not documented in four cases. Overall, 164 patients (65%) had second-line treatment, 75 (30%) third-line, 39 (15%) fourth-line, and 18 (7.1%) fifth-line treatment. The most frequent first-line therapy was SSA (*n* = 160, 63%), followed by PRRT (*n* = 33, 13%), everolimus (*n* = 31, 12%), capecitabine/temozolomide (CAPTEM, *n* = 13, 5%), platin/etoposide (*n* = 9, 4%), and other (*n* = 8, 3%). In the second line, PRRT was the most common therapy (*n* = 79, *n* = 48%), followed by everolimus (*n* = 27, 16%), CAPTEM (*n* = 15, 9%), and SSA (*n* = 13, 8%). PRRT (*n* = 20, 27%) or re-PRRT (*n* = 18, 24%) were also the most common third-line therapies, whereas everolimus (*n* = 8, 11%), CAPTEM (*n* = 8, 10%), and SSA (*n* = 6, 8%) were less common, see Fig. 1. The median time between first- and second-line treatment start was 18.6 months, 16.2 months between the second and third line, 14.3 months between the third and fourth line, and 12.5 months between the fourth and fifth line.

### Patient characteristics at different treatment lines

**Fig. 2:**
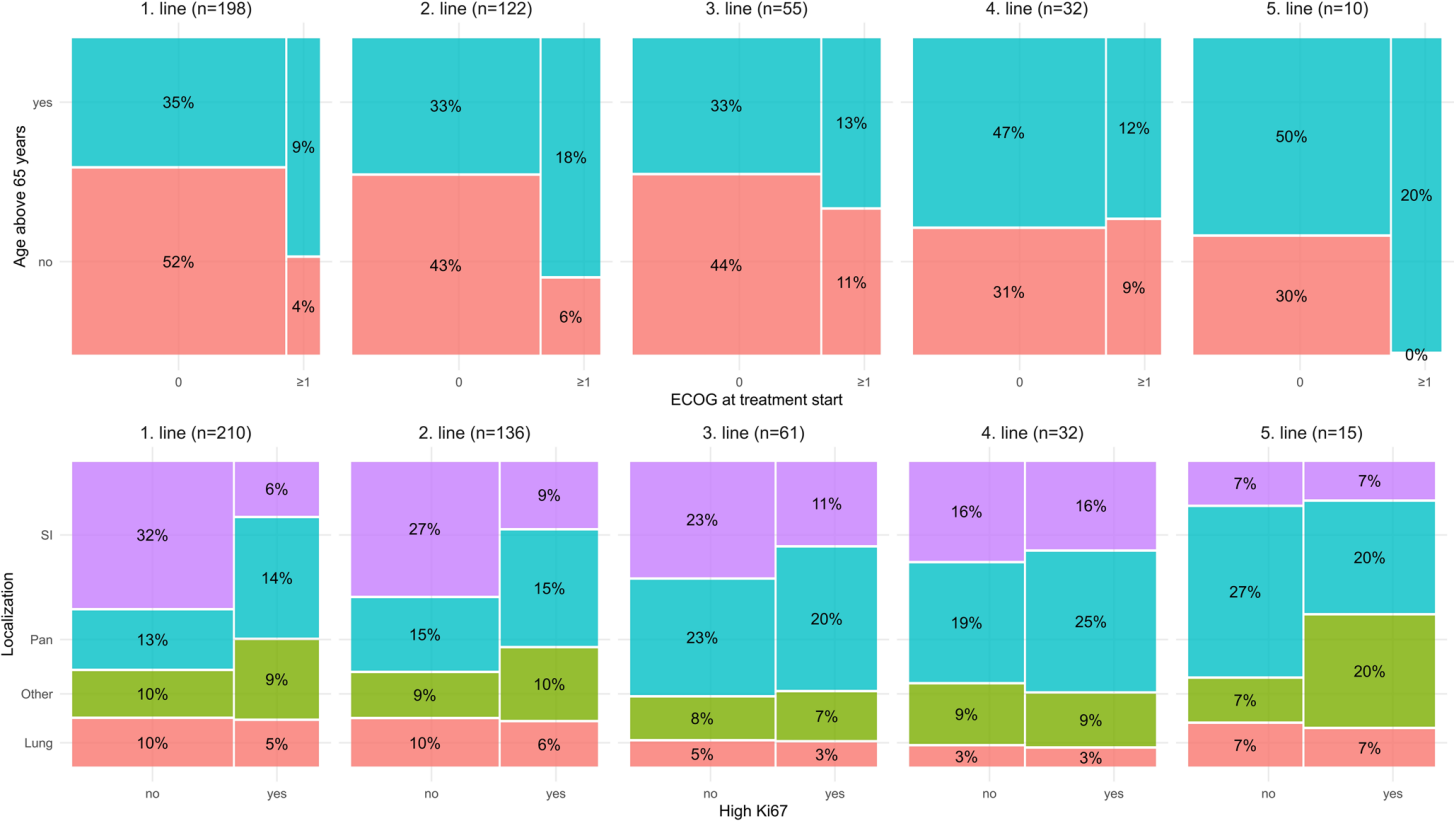
Patient characteristics for the respective treatment lines ( patients with missing values for ECOG and Ki-67 index were excluded)

Aside from the changing treatment patterns, the characteristics of patient subsets also differed between the individual treatment lines, see Fig. 2. As expected, the percentage of older (> 65 years of age at treatment start) patients with ECOG ≥ 1 increased from 9% (first line) to 12–20% (later lines), whereas the fraction of patients with ECOG 0 and age < 65 years decreased (52% to 30–44%). There were relatively more patients with pancreatic NET in later treatment lines, increasing from 14% (*n* = 29) and 13% (*n* = 27) for high (≥ 10%) and low (< 10%) Ki-67 index, respectively, in the first line to 20% (*n* = 12) and 23% (*n* = 14), respectively, in the third line. In contrast, patients with small intestinal NET were less frequently treated with fourth- or fifth-line systemic treatment (16% each and 7% each, respectively).

### Blood marker dynamics

**Fig. 3:**
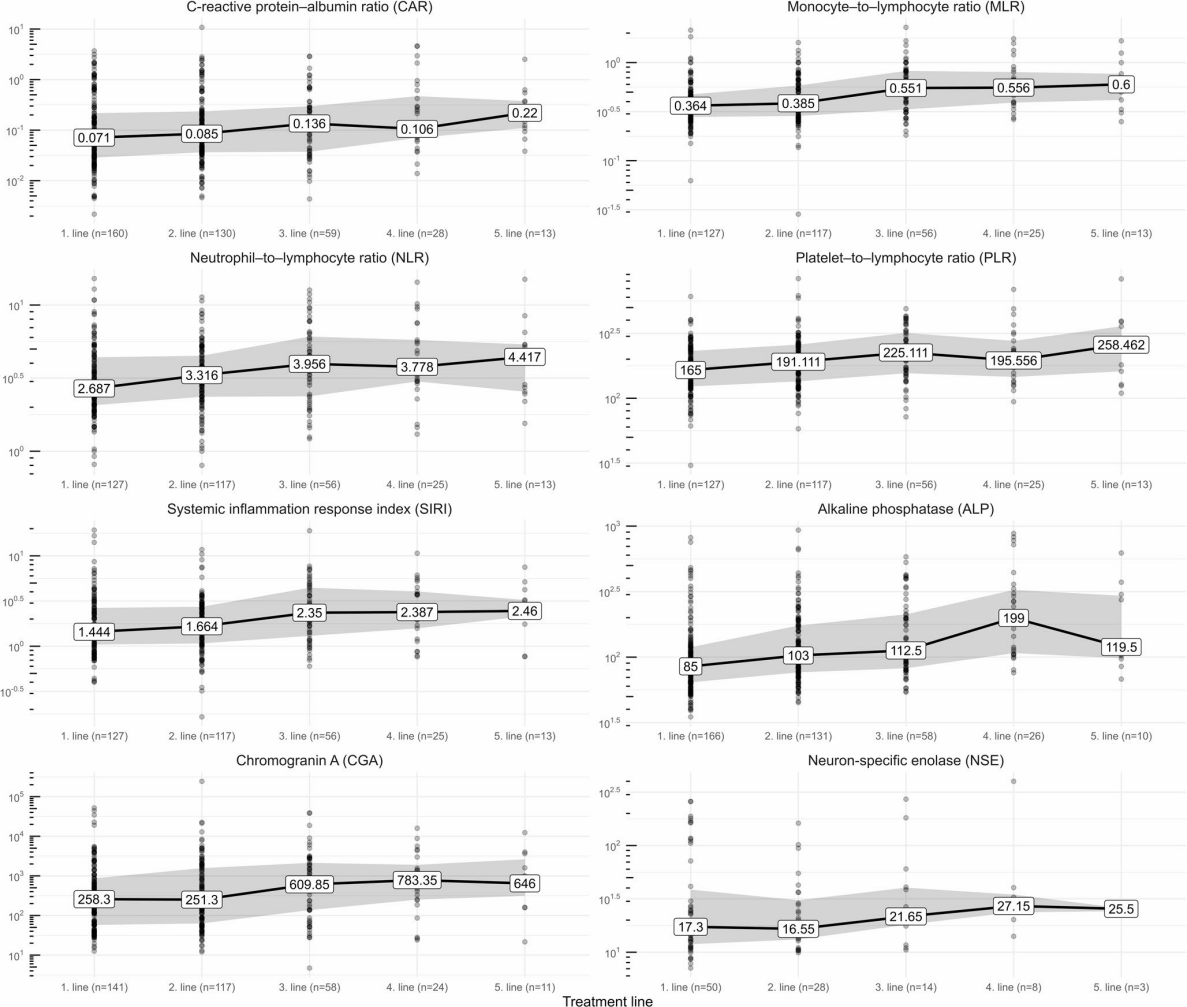
Blood marker dynamics with values (dots), inter-quartile ranges (ribbon), and medians (line) at start of the respective treatment line

With respect to the blood markers of interest in this study, there was a general trend to higher medians in later therapy lines, but the absolute number of measurements also decreased progressively, see Fig. 3. The median CAR increased from 0.07 (range: 0.002–3.70) in the first line to 0.14 (range: 0.004–2.89) in the third line. For MLR, the medians were 0.36 (range: 0.06–2.14) and 0.55 (range: 0.18–2.29), respectively. The median NLR was 2.69 (range: 0.81–15.17) at first line start and 3.96 (range: 1.22–12.67) at third line start, while the medians were 165.0 (range: 30.38–608.33) and 225.11 (range: 71.82–490.0), respectively, for PLR. The median SIRI rose from 1.44 (range: 0.4–19.29) to 2.35 (range: 0.6–18.97). Regarding ALP at first line start, the median was 85 U/l (range: 35–818 U/l) and at third line start 112.5 U/l (range: 54–583 U/l). The median CGA level increased from 258.3 ng/ml (range: 12.6–52020 ng/ml) to 609.85 ng/ml (range: 4.7–38750 ng/ml). Lastly, the median for NSE was 17.3 µg/l (range: 7.1–259 µg/l) before the first line and 21.65 µg/l (range: 10.5–272.0 µg/l) before the third line.

### Survival according to treatment lines

**Fig. 4:**
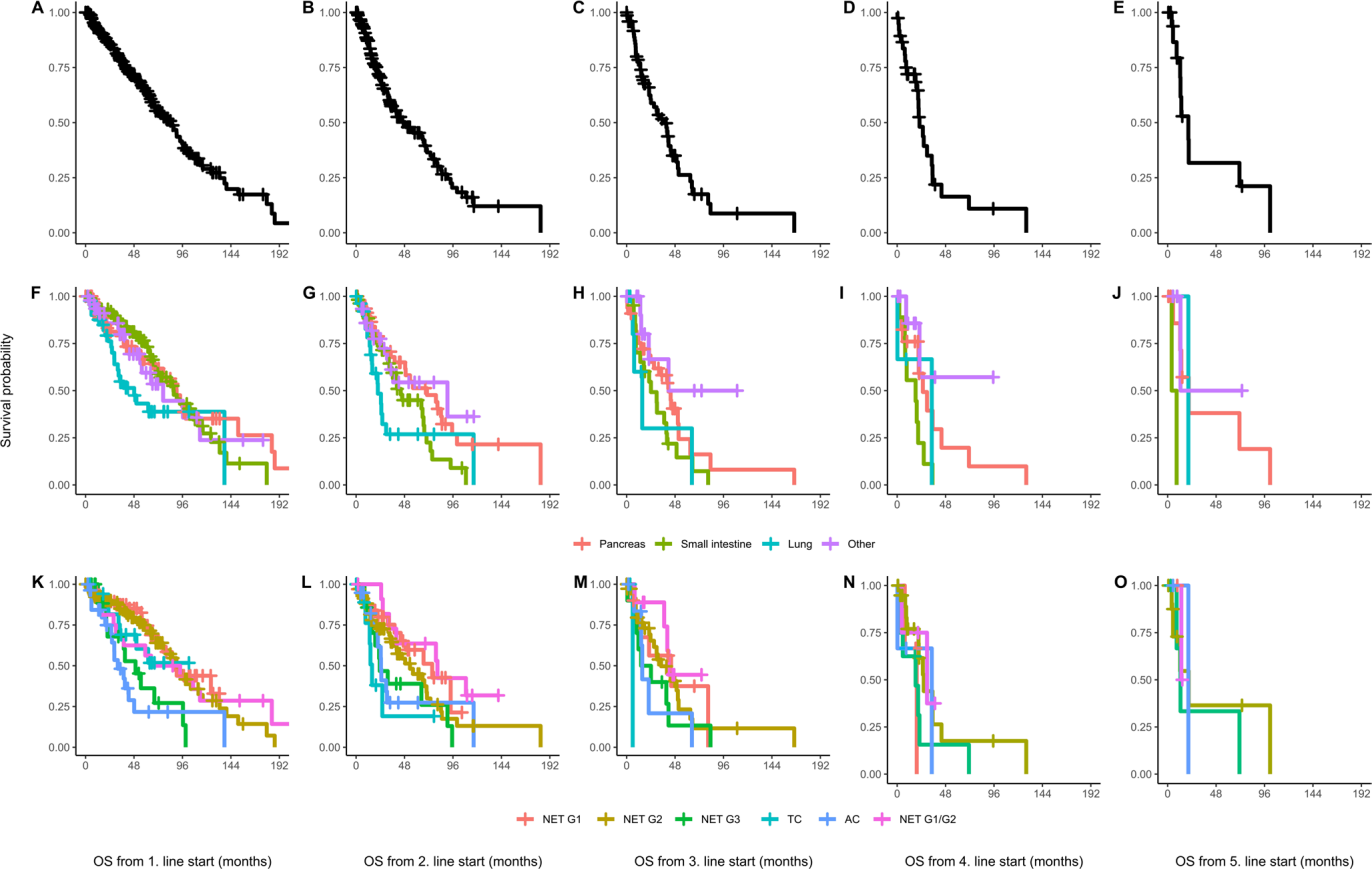
Kaplan–Meier plots of overall survival from first- to fifth-line start for the overall study population (A-E) and based on primary tumor site (F-J) and grading (K-O)

In the overall study population, the median OS from first line start was 84.3 months (95% CI 69.1–95.6 months), with the median follow-up being 68.3 months according to reverse Kaplan–Meier. As expected, the median OS from treatment start decreased with increasing LoT, from 84.3 months from first line start to 48.6 months (95% CI 34.3–69.8 months) from second line initiation and 37.2 months (95% CI 23.7–49.5 months) from third line start, see Fig. 4. The median OS was 22.2 months (95% CI 20.8–35.1 months) and 20.5 months (95% CI 12.4 – not calculable [NC] months) from fourth and fifth line, respectively. Compared to the median OS of 90.2 months (95% CI 69.4–NC months) from first line start for pancreatic NET, the median OS for the second line was 70.2 months (95% CI 48.6–100.0 months) and for the third line 41.5 months (95% CI 27.1–83.1 months). Similarly, the median OS was progressively shorter for small intestinal NET, with a median OS of 44.2 months (95% CI 33.0–67.5 months) and 24.1 months (95% CI 13.5–49.5 months), respectively, as compared to the 87.4 months (95% CI 69.1–113.2 months) from first line start. For lung NET, the median OS decreased from 48.1 months (95% CI 28.5–NC months) to 21.5 months (95% CI 15.8–NC months) and 15.5 months (95% CI 7.4–NC months), respectively.

Likewise, patients with NET G1 had a median OS of 87.4 months (95% CI 72.7–NC months) from first line start, 75.4 months (95% CI 44.2–NC months) from second line start, and 44.0 months (95% CI 13.5–NC months) from third line start. NET G2 showed a first-line median OS of 90.2 months (95% CI 79.2–116.1 months), a second-line median OS of 53.4 months (95% CI 40.2–73.7 months), and a third-line median OS of 34.2 months (95% CI 27.1–63.1 months). NET G3 was associated with a median OS of 49.5 months (95% CI 21.7–NC months), 23.4 months (95% CI 16.1–NC months), and 19.2 months (95% CI 10.0–NC months), respectively. Patients with typical carcinoids had a median OS of not reached, 15.8 months (95% CI 13.5–NC months), and 6.0 months (95% CI NC–NC months), respectively, while patients with atypical carcinoids had a median OS of 31.9 months (95% CI 26.1–NC months) from the first line, 24.6 months (95% CI 21.3–NC months) from the second line, and 15.5 months (95% CI 14.4–NC months) from the third line.

### Prognostic factors according to treatment lines

In univariate Cox regression analysis of general clinical characteristics, age at treatment start > 65 years (HR 2.62), ECOG at treatment start ≥ 1 (HR 3.04), histology of NET G3 (HR 2.58) or atypical carcinoid (HR 3.33), Ki-67 index ≥ 10% (HR 2.21), and NET of lung origin (HR 2.08) were negative prognostic factors, whereas sex was not a significant factor. Surgery and tumor functionality were removed from the Cox model due to a violation of the proportional hazards assumption. In multivariable analysis, age, ECOG, NET G3, and high Ki-67 remained statistically significant, see Table 2.

**Table 2:** Cox regression analysis of overall survival after first line start

Characteristic	Univariate				Multivariable		
	*N*	HR	95% CI	*p*-value	HR	95% CI	*p*-value
**Sex**	250						
*Female*		—	—		—	—	
*Male*		1.31	0.90, 1.90	0.2	1.04	0.65, 1.65	0.9
**Over 65 years at therapy start**	250						
*no*		—	—		—	—	
*yes*		2.62	1.79, 3.83	**< 0.001**	2.16	1.34, 3.49	**0.002**
**ECOG at therapy start**	198						
*0*		—	—		—	—	
*≥1*		3.04	1.78, 5.18	**< 0.001**	3.05	1.57, 5.92	**0.001**
**Grading**	250						
*NET G1/G2*		—	—		—	—	
*NET G3*		2.58	1.39, 4.78	**0.003**	2.22	1.01, 4.87	**0.047**
*TC*		1.37	0.63, 3.00	0.4	3.03	0.27, 33.9	0.4
*AC*		3.33	1.91, 5.78	**< 0.001**	4.96	0.59, 41.7	0.14
**Ki-67 index ≥ 10%**	210						
*no*		—	—		—	—	
*yes*		2.21	1.43, 3.42	**< 0.001**	1.81	1.01, 3.22	**0.045**
**Primary tumor site**	250						
*Pancreas*		—	—		—	—	
*Small intestine*		1.07	0.67, 1.70	0.8	1.21	0.68, 2.17	0.5
*Lung*		2.08	1.18, 3.69	**0.012**	0.43	0.04, 4.03	0.5
*Other*		1.14	0.63, 2.09	0.7	1.13	0.52, 2.45	0.8

CI, confidence interval. HR, hazard ratio

**Fig. 5:**
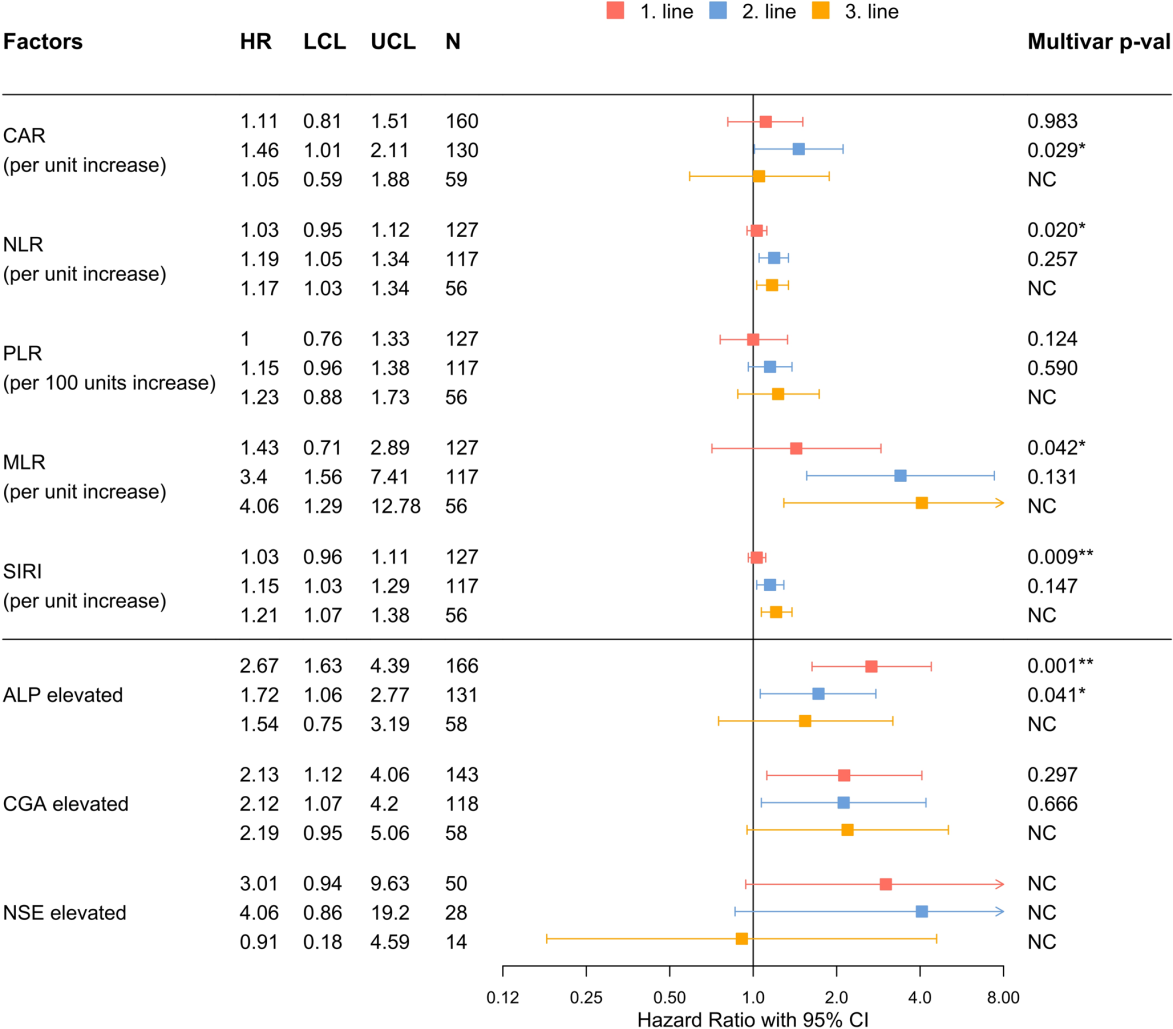
Forest plot comparing the prognostic impact of blood markers between different treatment lines according to univariate Cox regression analysis of overall survival from treatment start (hazard ratios and confidence intervals) and after covariate adjustment (multivariable-analysis p-value). HR, hazard ratio. LCL, lower confidence limit. UCL, upper confidence limit. N, number. NC, not calculated

Regarding the blood-based inflammation indices, none of the ratios proved to be a significant prognosticator in univariate Cox regression analysis from first line start, with, for instance, hazard ratios of 1.03 (NLR) and 1.43 (MLR) and confidence intervals that were crossing 1.0, see Fig. 5. However, in the second line, CAR, NLR, MLR, and SIRI were significantly associated with worse survival, that is, a 46% higher risk of death per unit increase for CAR, while the risk was higher by 19% per unit increase for NLR, by 240% for MLR, and by 15% for SIRI. Despite the smaller sample size (*n* = 56), NLR, MLR, and SIRI were also prognostically significant in the analysis for OS from third line initiation. After adjusting for the prognostically most relevant variables from Table 2, i.e., age, ECOG, and grading, NLR (HR 1.14, *p* = 0.02), MLR (HR 2.56, *p* = 0.042), and SIRI (HR 1.12, *p* = 0.009) emerged as significant regarding OS from first-line start, while CAR achieved statistical significance in the second line (HR 1.88, *p* = 0.029).

Lastly, the prognostic value of elevated levels of CGA, NSE, and ALP was evaluated, see Fig. 5. Elevated ALP indicated significantly worse survival from first line start with a HR of 2.67 and elevated CGA with a HR of 2.13, whereas elevated NSE (HR 3.01) failed to reach statistical significance. Furthermore, in the second line subset, ALP (HR 1.72) and CGA (HR 2.12) were also significantly associated with OS. After covariate adjustment, only elevated ALP levels were prognostic for OS from first-line (HR 2.49, *p* = 0.001) and second-line start (HR 1.82, *p* = 0.041).

## Discussion

In this study, patient subsets at the start of different treatment lines were evaluated regarding patient characteristics, treatment patterns, and the significance of prognostic factors, assessed at different points in the course of the disease. As NET patients are likely to receive many LoT throughout their NET disease and as the number of treatment options can be expected to increase in the future, mapping these dynamics over time is of interest. In particular, we wanted to explore whether clinical factors remained equally prognostic in advancing lines of treatment. In the overall cohort of 254 patients with metastatic NET, high age (> 65 years), ECOG ≥ 1, NET G3, and Ki-67 ≥ 10%, were negative prognostic factors in multivariable analysis of OS from first-line start. Apart from different treatment choices in later lines, the structure of patient subsets changed, with not only more older patients but relatively more pancreatic NET with higher Ki-67 index values in later treatment lines, which confirms the negative prognostic influence of this biological subgroup. CGA as well as ALP exhibited prognostic value for OS from first-line and second-line treatment start in univariate analysis, whereas blood-based inflammation markers generally had mixed prognostic significance (e.g., none of the studied ratios were significant regarding OS from first-line start in univariate analysis, but some were in multivariable analysis).

After a median follow-up of over 5.5 years, almost two-thirds (65%) of patients had started a second-line treatment and about one-third (30%) a third-line therapy. In clinical practice, the sequencing of available treatments represents a challenging issue, as the number of treatment options is increasing and as comparative data are currently lacking [36]. A relevant finding of this analysis is the dynamic change in patient characteristics and selected blood values when comparing patients receiving a late versus an early LoT. One might speculate on the underlying factors driving this development: Patients had higher age as they were in a higher treatment line, differed in tumor primary localization, and could potentially have had long-term toxicity from previous therapies or increased tumor load at an advanced stage. For example, patients with more rapidly growing NET may die earlier and only receive one treatment line or they may progress through several lines more quickly, whereas patients with indolent NET might remain years on first-line treatment. Although tumor load was not specifically measured, increasing disease burden could explain the increase in blood markers such as CAR, NLR, ALP, or CGA over time. Hence, different LoT subpopulations are not directly comparable.

In detail, we presented OS estimates for different LoT subpopulations with a certain primary tumor origin or tumor grade and observed progressively shorter OS with each additional treatment line, from 84.3 months from first-line start and 48.6 months from second-line start to 37.2 months from third-line and 22.2 months from fourth-line start. For reference, based on epidemiologic data on distant well- to moderately differentiated NET in the SEER registry, median OS was 65 months for jejunoileal NET, 27 months for pancreatic NET, and 17 months for lung NET [37]. In larger retrospective studies of metastatic NET, a median OS of about 121 months (10.1 years) was reported for small bowel NET [38], 86 months for pancreatic NET [22], and 80.9 months for lung NET [9]. With regard to survival measured from LoT start, median OS was about 84 months in untreated midgut NET in PROMID [25], 36.3–48.0 months in SSA-pretreated midgut NET in NETTER-1 [39], and 19.7–21.9 months in extrapancreatic NET receiving cabozantinib after a median of two prior LoT (excluding SSA) in CABINET [40]. Thus, these survival data are largely comparable to our single-center real-world data.

The blood-based inflammation indices did not demonstrate a consistent prognostic utility for OS in our study. Interestingly, there was significant prognostic potential in the second (CAR, NLR, MLR, and SIRI) or third line (NLR, MLR, and SIRI) in univariate analysis and in the first line (NLR, MLR, and SIRI) and second line (CAR) after adjusting for other prognostic factors. Whilst this could be random or for statistical reasons (scores were modeled as continuous rather than binary variables), it might also indicate that the interplay between systemic inflammatory processes and survival may be more complex in NET. Possible molecular links between inflammation and NET are circulating proinflammatory cytokines [41] and angiogenic factors [42] and warrant further investigation regarding their prognostic impact. In addition, this could also reflect the heterogeneity of NET in terms of varying disease trajectories and different treatment patterns. In comparison, a systematic review and meta-analysis of 32 studies on inflammation-related blood markers in NEN showed a significant relationship between OS and NLR (HR 2.09) as well as PLR (HR 1.79), while higher CRP levels alone, for instance, were associated with a HR of 2.88 (all *p* < 0.001) [17]. Furthermore, higher PLR and CRP were related to significantly worse progression-free survival (PFS) in this meta-analysis [17]. A study worth mentioning in this context is an exploratory analysis of randomized trial data from CLARINET (lanreotide autogel versus placebo), which showed no prognostic value of NLR with regard to PFS [43]. Further evidence seems necessary before these indices can be recommended for clinical routine.

With regard to the tumor markers CGA and NSE, only few measurements were available for advanced LoT, which might explain the lack of statistical significance for OS from third-line start, for instance. Furthermore, it is noteworthy that ALP was the only variable that was significant in the first and second LoT subpopulation both in univariate analysis and after adjusting for other prognostic factors. In contrast, CGA showed no independent prognostic value, which was also the case in an earlier analysis involving 137 patients [16].

Limitations of this study included the restricted sample size, especially in later treatment lines, with broad confidence intervals for the hazard ratios of certain factors. Considering the exploratory nature of this analysis, it should be noted that many variables were evaluated for prognostic significance without adjusting for multiple testing. The lack of sample size planning has several implications, including uncertain statistical power and higher sampling variability. Furthermore, the study cohort and particularly the treatment patterns were heterogeneous, and results may have been influenced by unobserved confounding factors. The retrospective single-center design is another constraint of this analysis, and a prospective multi-center validation should be encouraged.

In conclusion, this analysis demonstrates a longitudinal approach to risk factor analysis in NET. Patient characteristics and treatment patterns were varying at the start of the different LoT. This study confirmed the strong prognostic effect of age, ECOG, grading, and Ki-67 index in NET. Moreover, ALP and CGA were prognostic at least in univariate analysis. In contrast, blood-based inflammation indices showed inconsistent prognostic utility in our study, which is not conclusive and might underscore that inflammation in NET is a complex and dynamic process worth further investigation.

## Data Availability

Enquiries for further data can be directed to the corresponding author.

## Declarations

### Statement on the use of artificial intelligence

Artificial intelligence (AI)-based technologies were used in the conduct of this study. ChatGPT version 4 and newer (OpenAI, Inc.; 2023-2026; https://chatgpt.com/) was used to support R programming for statistical analyses and data visualization. The author(s) reviewed all outputs of this AI-assisted tool/service and edited the content as needed and take(s) full responsibility for the accuracy and integrity of this work.
